# Label-Free Proteomics of Oral Mucosa Tissue to Identify Potential Biomarkers That Can Flag Predilection of Precancerous Lesions to Oral Cell Carcinoma: A Preliminary Study

**DOI:** 10.1155/2023/1329061

**Published:** 2023-02-01

**Authors:** Vipra Sharma, Sabyasachi Bandyopadhyay, Kapil Sikka, Aanchal Kakkar, Gururao Hariprasad, Sundararajan Baskar Singh

**Affiliations:** ^1^Department of Biophysics, All India Institute of Medical Sciences, New Delhi 110029, India; ^2^Proteomics Lab, Central Core Research Facility, All India Institute of Medical Sciences, New Delhi 110029, India; ^3^Department of Otorhinolaryngology, All India Institute of Medical Sciences, New Delhi 110029, India; ^4^Department of Pathology, All India Institute of Medical Sciences, New Delhi 110029, India

## Abstract

Oral squamous cell carcinomas are mostly preceded by precancerous lesions such as leukoplakia and erythroplakia. Our study is aimed at identifying potential biomarker proteins in precancerous lesions of leukoplakia and erythroplakia that can flag their transformation to oral cancer. Four biological replicate samples from clinical phenotypes of healthy control, leukoplakia, erythroplakia, and oral carcinoma were annotated based on clinical screening and histopathological evaluation of buccal mucosa tissue. Differentially expressed proteins were delineated using a label-free quantitative proteomic experiment done on an Orbitrap Fusion Tribrid mass spectrometer in three technical replicate sets of samples. Raw files were processed using MaxQuant version 2.0.1.0, and downstream analysis was done via Perseus version 1.6.15.0. Validation included functional annotation based on biological processes and pathways using the ClueGO plug-in of Cytoscape. Hierarchical clustering and principal component analysis were performed using the ClustVis tool. Across control, leukoplakia, and cancer, L-lactate dehydrogenase A chain, plectin, and WD repeat-containing protein 1 were upregulated, whereas thioredoxin 1 and spectrin alpha chain, nonerythrocytic 1 were downregulated. Across control, erythroplakia, and cancer, L-lactate dehydrogenase A chain was upregulated whereas aldehyde dehydrogenase 2, peroxiredoxin 1, heat shock 70 kDa protein 1B, and spectrin alpha chain, nonerythrocytic 1 were downregulated. We found that proteins involved in leukoplakia were associated with alteration in cytoskeletal disruption and glycolysis, while in erythroplakia, they were associated with alteration in response to oxidative stress and glycolysis across phenotypes. Hierarchical clustering subgrouped half of precancerous samples under the main branch of the control and the remaining half under carcinoma. Similarly, principal component analysis identified segregated clusters of control, precancerous lesions, and cancer, but erythroplakia phenotypes, in particular, overlapped more with the cancer cluster. Qualitative and quantitative protein signatures across control, precancer, and cancer phenotypes explain possible functional outcomes that dictate malignant transformation to oral carcinoma.

## 1. Introduction

Cancers of the lip and oral cavity are the most prevalent cancers worldwide, with a global incidence of more than 370,000 new cases and 177,000 deaths, annually, in 2020 with considerable geographic and environmental etiological differences [1]. Oral squamous cell carcinoma (OSCC) accounts for 90% of oral neoplasms [2]. These arise from the mucosal epithelium lining of the oral cavity, pharyngeal regions, and salivary glands [3]. The etiological factors range from exogenous factors such as smoking, tobacco consumption, human papillomavirus infection, autoimmune inflammation, malnutrition, and inherited genetic aberrations [4]. These factors may act separately or synergistically attributing to a significantly increased risk of oral cancer [5]. At a molecular level, tobacco induces a generation of reactive oxygen species (ROS) and reactive nitrogen species (RNS) either directly or through activation of inflammatory cascade leading to the development of chronic oxidative stress conditions [6].

Although OSCC can develop de novo apparently in normal mucosa, most of them are preceded by morphologically altered tissue in the form of precancerous lesions on the oral mucosa [7]. The two main types of oral premalignant lesions that manifest clinically as leukoplakia and erythroplakia are at different propensities of developing into carcinoma [8]. Leukoplakia is a clinical term for a white lesion in the oral mucosa that cannot be scraped off, whereas erythroplakia is a fiery red soft velvety lesion. These two lesions are very distinct and cannot be attributed clinically or histologically as another definable lesion [9, 10].

Erythroplakias are rarer lesions with a low prevalence between 0.02% and 0.83% but much more likely to progress to SCC at a transformation rate ranging from 14% to 50% [4, 11]. Leukoplakias, on the other hand, occur with a global prevalence of 2-3% but are at less risk with a transformation rate of 0.13% to 34% [11, 12]. The current gold standard for diagnosis of premalignant or malignant lesions is histopathological evaluation to determine the degree of severity of suspicious lesions to assess the transformation risk of oral premalignant lesions [13]. Recently, Fourier transform infrared (FTIR) imaging integrated with machine learning found biochemical differences in addition to existing histopathological diagnostic processes and provide oral cancer discrimination and a novel oral epithelial dysplasia stratification strategy identifying heterogeneity between intra-samples and inter-samples of the oral epithelium [14].

Genomic findings reported an accumulation of copy number changes and point mutations resulting in oral cancer which is a gradual random accumulation initiated at the precancerous stage rather than a dramatic single event [15]. Transcriptomic profiling studies of gingivobuccal oral cancer (OSCC-GB) and precancer identified genes specific to pathways such as ECM–receptor interaction, cytokine−cytokine receptor interaction, focal adhesion, cell cycle, and PI3K-Akt signaling that correlated with dysregulation of the underlying molecular mechanisms in OSCC-GB, therefore enhancing the risk of transformation from precancer to frank oral cancer [16].

Quantitative proteomic-based approaches have been used to identify multiple biomarkers that can discriminate premalignant lesions from normal mucosa. In one study, stratifin (SFN), 14-3-3 zeta (YWHAZ), and hRNPKs (heterogeneous nuclear ribonucleoprotein K) have been identified where direct interaction between three biomarkers have been reported for their role in inflammation, signaling, proliferation, and cancer [17]. hRNPK is shown to be expressed in early stages of oral leukoplakia as a malignant transformation-related protein [18]. In another study, solute carrier family 3 member 2 (SLC3A2), S100 calcium-binding protein A2 (S100A2), and interleukin-1 receptor antagonist protein (IL1RN) in saliva have been used to distinguish premalignant from malignant oral conditions [19]. However, these studies were on a small cohort, requiring validation on a larger sample size.

At our lab, we have been involved in the identification of potential biomarker candidates in various cancer phenotypes [20, 21]. This study is aimed at identifying protein signatures using label-free quantitative proteomics that can explain the transformation of oral premalignant lesions into squamous cell carcinoma.

## 2. Methodology

### 2.1. Ethics, Patient Screening, and Sample Collection

**Figure 1:**
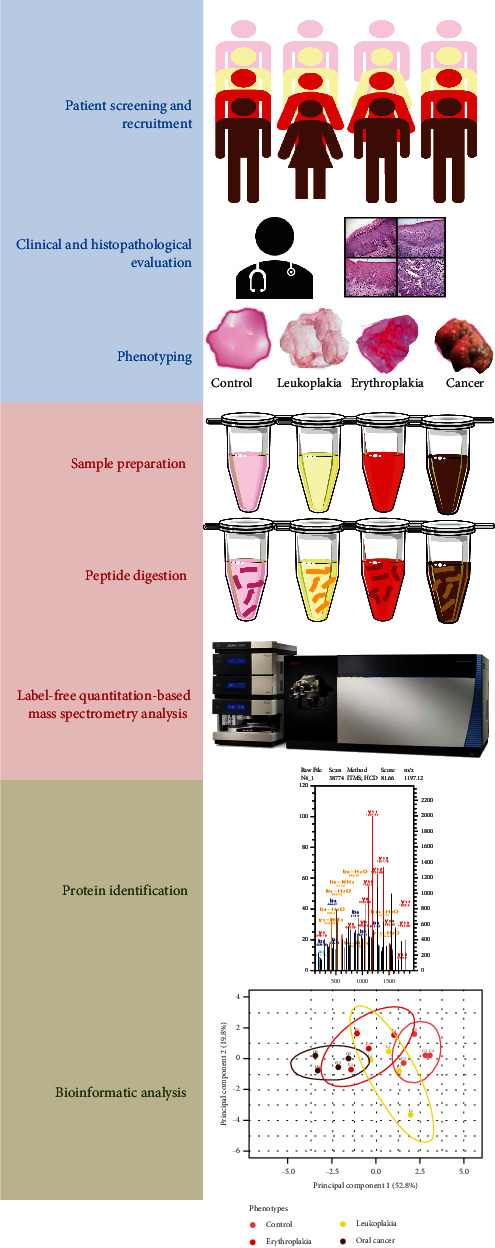
Label-free quantification proteomic analysis workflow. Sixteen biological replicates with suspected lesions were recruited. Disease phenotypes, control, leukoplakia, erythroplakia, and cancer, were assigned after clinical and histopathological evaluations. Oral mucosal tissues were processed for protein isolation, trypsin digestion, and desalting. Peptides were subjected to mass spectrometric analysis, and the generated raw files were analyzed for protein identification. Results were validated via bioinformatic analysis.

The study was approved by the Institute Ethics Committee at the All India Institute of Medical Sciences, New Delhi, India (Ref. no. IECPG-370/26.08.2020). A brief overview of the methodology is provided in Figure 1. All procedures were conducted by the guidelines of the Helsinki Declaration. Sixteen patients, four for each phenotype, were recruited for our study. Patients with leukoplakia, erythroplakia, and oral squamous cell carcinoma were screened based on the clinical presentation of patients at the Out-Patient Department of Otorhinolaryngology. Written informed consent was collected from each patient enrolled. Tissue from 5 mm away from the leukoplakic lesion was collected for consideration as cancer controls. Biopsies were taken from the suspected mucosal lesions and washed thoroughly with 1x PBS (pH 7.4) to remove blood from the specimen. Part of this sample was sent to the Department of Pathology for histopathological analysis, and the rest of the sample was stored at -80°C for proteomic experiments at the Proteomics Facility.

### 2.2. Patient Inclusion and Exclusion Criteria

Patients with lesions on oral buccal mucosa were included in the study. Patients who had received neoadjuvant chemotherapy or radiotherapy or had coexisting morbidities and infections were excluded from the study.

### 2.3. Histopathology

Tissue sent to the Department of Pathology was collected in 10% formalin and embedded in paraffin for histopathological analysis. 4 *μ*m thick paraffin-embedded sections were deparaffinized with three subsequent washes in xylene and then rehydrated by giving subsequent washes in 100% ethanol, 90% ethanol, 70% ethanol, and distilled water. Sections were stained with hematoxylin and washed in running water for 5 minutes, then stained in eosin solution for two minutes, and then rinsed with 95% ethanol. They were then subjected to 95% ethanol and 100% ethanol for two minutes, twice. Tissue sections were mounted with a drop of distyrene plasticizer xylene (DPX) after exposure to xylene. The hematoxylin and eosin-stained slides were examined under a microscope, and specimens were reported as per the World Health Organization 2017 classification for the histopathological diagnosis of oral epithelial dysplasia and squamous cell carcinoma. Annotation of collected samples as leukoplakia, erythroplakia, or oral squamous cell carcinoma was based on both clinical features and histopathology.

### 2.4. Protein Extraction

Tissue samples were minced, and the proteins were solubilized in a lysis buffer containing 8 M urea, 2 M thiourea, and 4% 3-[(3-cocamidopropyl)dimethylammonio]1-propanesulfonate (CHAPS). Tissue was homogenized by a handheld homogenizer followed by ultrasonication in a water bath at 45 Hz frequency and 90 W power for 20 min. The lysate was centrifuged at 12,000 rpm for 15 minutes at 4°C, and the supernatant was collected. Detergents and lipids were removed from the extracted protein using a 2D clean-up kit as per the manufacturer's protocol. The pellet was reconstituted in 8 M urea/50 mM Tris-HCl (pH 8). The protein concentration was estimated using a Bradford assay.

### 2.5. In-Solution Tryptic Digestion, Desalting, and Peptide Quantification

20 *μ*g of protein from each sample was reduced with 25 mM DTT for 30 min at 37°C and alkylated with 55 mM iodoacetamide for 20 min in the dark at room temperature. The reaction was diluted with three volumes of 50 mM Tris-HCl (pH 8). Trypsin (mass spectrometry grade, Promega, USA) which was reconstituted in 50 mM ammonium bicarbonate in a 1 : 20 ratio was added and incubated at 37°C for 16 h. Subsequently, the reaction was quenched by 10% TFA in ammonium bicarbonate. Peptides were desalted using C18 zip tips (Sigma-Aldrich, USA) of 0.6 *μ*l bed volume following the manufacturer's protocol, lyophilized by vacuum centrifugation, reconstituted in 0.1% (*v*/*v*) FA in LC-MS/MS grade water (Sigma-Aldrich, USA), and quantified as per the Scopes method. Peptide concentration was normalized as 0.1 *μ*g/*μ*l.

### 2.6. Mass Spectrometry Analysis

Four biological and three technical replicates were run on an Orbitrap Fusion Tribrid mass spectrometer (Thermo Fisher Scientific, USA) coupled with an ultrapressure EASY-nLC 1200 nano-LC system (Thermo Fisher Scientific, USA). The mobile phase consisted of LC-MS/MS grade water with 0.1% formic acid as mobile phase A (loading buffer) and 0.1% formic acid/80% acetonitrile as mobile phase B (loading buffer). Digested and desalted peptides of each biological sample were reconstituted in mobile phase A and 1 *μ*g injected onto a trap column, Acclaim PepMap 100 C18 (75 *μ*m × 2 cm, 3 *μ*m, 100 Å; Thermo Fisher Scientific) at a flow rate of 300 nl/min. Peptides were washed isocratically with a loading buffer (0.1% formic acid) for 45 minutes to remove excess salt, and then, retained peptides were resolved on an analytical column, Acclaim PepMap 100 C18 (75 *μ*m × 15 cm, 2 *μ*m, 100 Å; Thermo Fisher Scientific) before coupling to the mass spectrometer. Gradient elution was initiated using 5% elution buffer and was ramped up with a linear increase rate of 8% for 5 minutes, 60% for 110 minutes, and 95% for 2 minutes. Gradient elution was held in a 95% elution buffer for the next 5 minutes before being reequilibrated in a 5% elution buffer for 20 minutes. During the LC-MS/MS experiment, blank (loading buffer) and standard samples (HeLa digest) were included in between the experimental sample runs to wash the column and to check the QC/QA over the run, respectively. Each biological sample was analyzed thrice as technical triplicates to increase quantification confidence and reproducibility.

The mass spectrometer was operated in the data-dependent acquisition (DDA) mode. The full MS spectra were acquired in the positive ionization mode with an ion spray voltage of 2100 V and m/z ratio of 350-2000 Da, with a 50-millisecond injection time. For precursor/peptide isolation in the MS1 level, a quadrupole ion filter and orbitrap mass analyzer were used with the resolution setting of 60,000 (at 200 m/z) and AGC target of 1e6. The top 20 precursors were selected and fragmented for product ion isolation (MS2 or MS/MS) via a linear ion trap with a resolution setting of ~30,000 (at 200 m/z) and an AGC target of 1e5. For DDA, advanced “rolling collision energy” was applied for subsequent MS/MS scans with the normalized high-energy collision-induced dissociation (HCD) fragmentation energy set to 30%.

### 2.7. Validation: Data Processing and Downstream Bioinformatic Analysis

Raw files were processed with MaxQuant version 2.0.1.0 (Max Planck Institute of Biochemistry, Munich, Germany), and tandem mass spectra were searched with the Andromeda search algorithm against the UniProt-reviewed human database (FASTA files downloaded in June 2021) [22]. Searches were performed with full tryptic specificity, maximum 2 missed cleavages, precursor ion tolerance of 20 ppm in the first search used for recalibration, followed by 7 ppm for the main search and 0.5 Da for fragment ions. Carbamidomethylation of cysteine was set as a fixed modification, and methionine oxidation and protein N-terminal acetylation were set as variable modifications. Peptide spectral matches were made against a target-decoy human reference proteome database downloaded from UniProt. The required false discovery rate (FDR) was set to 1% for both peptide and protein levels, and the minimum required peptide length was set to seven amino acids. Proteins were quantified and normalized using MaxLFQ with a label-free quantification (LFQ) minimum ratio count of 2. LFQ intensities were calculated using the match between runs feature, and MS/MS spectra were required for LFQ comparisons. The downstream proteins and statistical analyses were performed with the Perseus software version 1.6.15.0 (Max Planck Institute of Biochemistry, Munich, Germany) [23]. Only those proteins that were present in all technical replicates of 16 biological replicates were taken into account for relative comparison. Protein intensity values were log_2_-transformed followed by sample normalization with median subtraction and *z*-scored feature normalization. A multiple-sample test using analysis of variance (ANOVA) was performed on phenotypic groups using the permutation-based false discovery rate (FDR) approach, preserving technical grouping in randomizations at 0.05 FDR. Proteins found significant after ANOVA and showing linear expression across control, leukoplakia/erythroplakia, and cancer were graphed using SigmaPlot version 14.0 (Systat Software, San Jose, CA). Fold change was determined for cancer phenotypes with precancerous phenotypes. Hierarchical clustering and principal component analysis (PCA) using ClustVis of averaged technical replicates were performed on 5 protein sets specifically involving leukoplakia and erythroplakia [24]. Both rows and columns were clustered using correlation distance and average linkage. Principal component analysis was additionally performed on 8 proteins present in either leukoplakia or erythroplakia. Unit variance scaling was applied to rows; singular value decomposition with imputation was used to calculate principal components. ClueGO v2.5.8 + CluePedia v1.5.8 plug-in (downloaded on May 2021) on Cytoscape version 3.9.0 was used for Gene Ontology biological function and KEGG pathway identification by applying a two-sided hypergeometric test with a *p* value cut-off of <0.05 [25].

## 3. Results and Discussion

### 3.1. Clinical Phenotyping and Sample Annotation

Sixteen patients were recruited in the discovery phase of the study, with four of them in each of the phenotypic groups of nonneoplastic control, leukoplakia, erythroplakia, and oral cancer. The clinical profile of these patients has been provided in Table 1. Detailed demographic features of the patient cohort are highlighted in Table 2. Most of these patients were male. This is consistent with the patient cohort of precancerous and cancerous oral lesions [26, 27]. The mean age of patients with cancer was found to be more than that of patients with precancerous lesions. Majority of the patients in the study had a history of tobacco consumption, which is a major etiological factor in patients who develop cancerous oral lesions [28]. Patients were first screened based on the clinical features, and the biopsy specimen that was procured from them was sent for histopathological evaluation, for sample annotation. As normal oral mucosa could not be procured due to ethical concerns, normal-looking mucosal tissue adjacent to leukoplakic lesions were annotated as cancer controls. Most of these tissue samples were found to be having epithelial hyperplasia. Histopathological profiling for all the phenotypes of cancer control, leukoplakia, erythroplakia, and oral squamous cell carcinoma is shown in Supplementary Figure 1.

### 3.2. Protein Profiling

**Figure 2:**
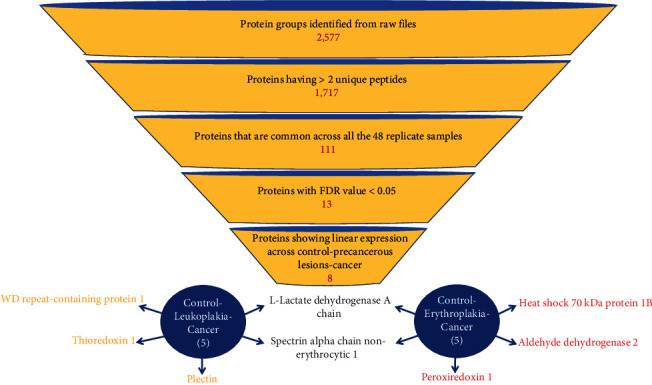
Protein filter criterion defined for selection of differentially expressed proteins in disease phenotypes.

**Figure 3:**
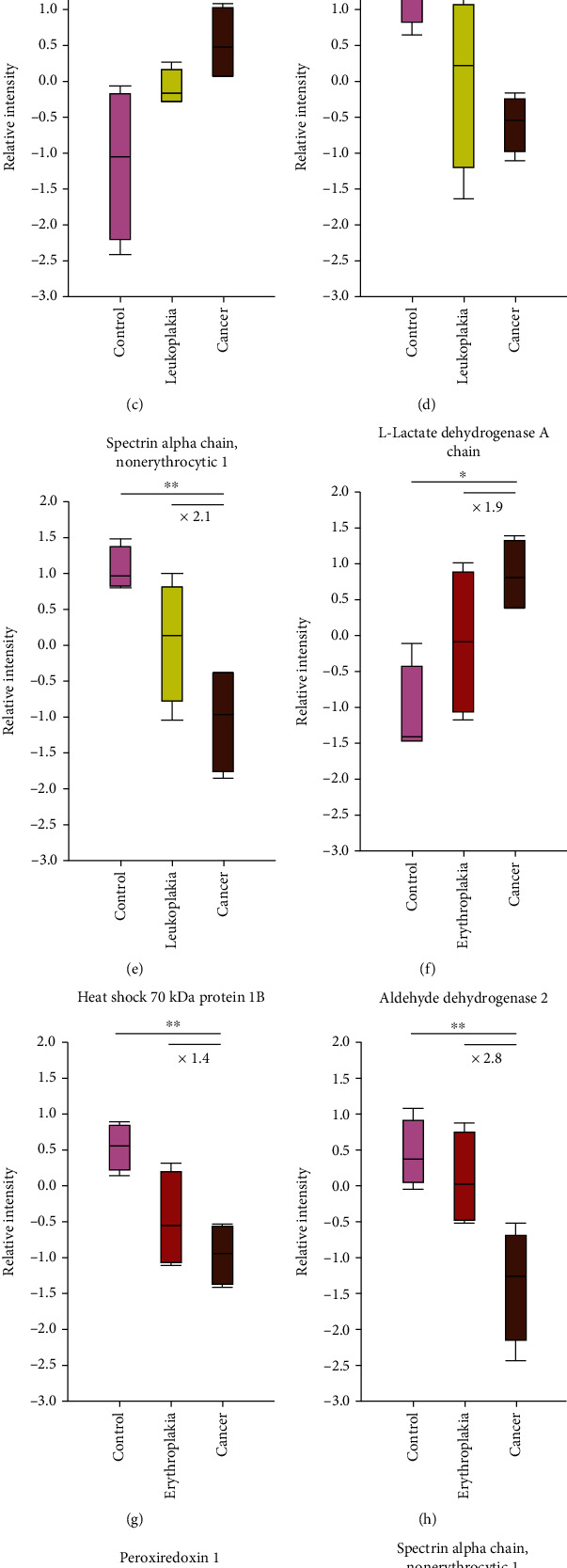
Expression level change of differentially expressed proteins. (1) Increased ratio of cancer/leukoplakia: (a) L-lactate dehydrogenase A chain, (b) WD repeat-containing protein 1, and (c) plectin; (2) decreased ratio of cancer/leukoplakia: (d) thioredoxin 1 and (e) spectrin alpha chain, nonerythrocytic 1; (3) increased ratio of cancer/erythroplakia: (f) L-lactate dehydrogenase A chain; and (4) decreased ratio of of cancer/erythroplakia: (g) heat shock 70 kDa protein 1B, (h) aldehyde dehydrogenase 2, (i) peroxiredoxin 1, and (j) spectrin alpha chain, nonerythrocytic 1. ^∗^ denotes *p* value < 0.05 and ^∗∗^ denotes *p* value < 0.01.

Mass spectrometric analysis of 48 replicates of buccal mucosa samples collectively resulted in identification of 2577 protein groups and 21,589 unique peptides. Proteins that were identified by site, reverse decoys, and potential contaminants were removed. Proteins having unique peptides >2 were selected for subsequent analysis. This resulted in 1717 proteins present in any of the samples and 111 proteins common in all 48 samples. Of these, 13 proteins fulfilled the statistical analysis criterion by ANOVA. There were 8 proteins that showed a linear proportionality across control, precancer, and cancer phenotypes. There were five differentially expressed proteins across phenotypic conditions of the control, leukoplakia, and cancer and five differentially expressed proteins across phenotypic conditions of the control, erythroplakia, and cancer. The systemic filter criteria that resulted in the final identification of 8 proteins that varied linearly across the three phenotypic groups are illustrated in Figure 2. The abundance-based quantification that is reflected as protein intensities for the eight proteins across the phenotypes is shown in Figure 3.

### 3.3. Differentially Expressed Proteins across the Three Clinical Phenotypes

Differentially expressed proteins and their functional relevance in this study is highlighted in Tables 3 and 4 and briefly discussed below.

#### 3.3.1. L-Lactate Dehydrogenase A (LDHA) Chain

LDH enzyme regulates glycolysis by catalyzing the reversible conversion of pyruvate to lactate under anaerobic conditions, replenishing NAD^+^ [29]. Of the two chains, the A chain of LDH that is differentially expressed is more efficient in catalyzing the conversion of pyruvate to lactate [30]. Unlike normal cells that employ the use of glycolysis and lactic fermentation for ATP production only under anaerobic conditions, cancer cells show a preference for glycolysis rather than oxidative phosphorylation even under normoxic conditions which is termed the Warburg effect [31]. This unique metabolic switch provides fast ATP generation to meet energy requirements of proliferating tumor cells [32]. Upregulation of LDH has been previously seen in erythroleukoplakia, leukoplakia, and verrucous lesion along with different grades of head and neck squamous cell carcinoma [33]. In our study, we found upregulation of LDHA in precancerous lesions and furthermore in cancer. The LDHA expression in cancer is 1.9 times higher compared to erythroplakia and 1.4 times higher compared to leukoplakia. This higher fold change observed in cancer with respect to erythroplakia compared to the leukoplakia phenotype is a plausible reason for the higher predilection of erythroplakia to cancer.

#### 3.3.2. Aldehyde Dehydrogenase 2 (ALDH2)

ALDH2 is a cellular detoxification enzyme that oxidizes endogenous aldehydic products arising from lipid peroxidation under oxidative stress [34]. In our study, we found a lower expression of ALDH2 in cancer compared to erythroplakia. This type of downregulation of ALDH2 in few other cancers as has been attributed to the acetaldehyde accumulation-driven cell redox status, promoting the AMP-activated protein kinase-driven glycolysis pathway to generate more ATP [35].

#### 3.3.3. Thioredoxin (TXN) 1 and Peroxiredoxin 1 (PRDX1)

An imbalance between the production and elimination of reactive oxygen species (ROS) gives rise to oxidative stress. Thioredoxin 1 together with peroxiredoxins constitutes the thioredoxin-peroxidase system that scavenges peroxides to protect cells from ROS-induced damage [36]. In our study, we found that levels of thioredoxin 1 and peroxiredoxin 1 were downregulated in cancer as compared to those of leukoplakia and erythroplakia, respectively. A lower level of salivary TXN has also been reported in patients with ulcerative SCC compared to those with leukoplakia and erythroplakia [37]. Reduced levels of thioredoxin and other antioxidants react with ROS contributing to the characteristic increased levels of oxidative stress in cancer cells [38]. Oxidative stress induces DNA mutation/DNA damage causing genome instability that marks the initiation and progression of cancer, promoting cellular proliferation, angiogenesis, and apoptosis [39].

#### 3.3.4. Heat Shock 70 kDa Protein 1B (HSPA1B)

The HSPA1B protein is a member of the heat shock protein 70 family that encodes a 70 kDa heat shock protein, also known as heat shock protein 70-2 (HSP70-2). HSP70 is a stress-inducible molecular chaperone with key roles that involve polypeptide refolding and degradation [40]. In our study, the HSPA1B expression was found to be decreased in cancer compared to erythroplakia. High expression of HSPA1B is known to enhance the resistance of cells to apoptosis-inducing proliferation and survival of cells [41]. Therefore, a higher expression of HSPA1B in erythroplakia is indicative of the transformation potential of a lesion to turn cancerous.

#### 3.3.5. Plectin (PLEC)

Plectin acts as a cytolinker that maintains the integrity of the cytoskeleton interacting with intermediary filaments and anchoring them at peripheral cell junctions, intracellular structures, and organelles [42]. In our study, we found a 1.5-fold change high expression of plectin in cancer compared to leukoplakia. Upregulation of plectin has been associated with proliferation, migration, and invasion in head and neck squamous cell carcinoma [43]. As compared to clinical samples of oral fibrous hyperplasias and dysplasias which are histological grades of precancerous lesions, the levels of plectin were found to be significantly higher in OSCCs and also in cell lines [44].

#### 3.3.6. Spectrin Alpha Chain, Nonerythrocytic 1 (SPTAN1)

SPTAN1 belongs to a family of cytoskeletal scaffolding proteins. This along with nonerythroid spectrin II (SPTBN1) interacts with different structural and regulatory proteins contributing to cell adhesion and migration [45]. In our study, we found the downregulation of SPTAN1 expression in oral cancer compared to precancerous lesions. Knockdown of SPTAN1 in cancer cell lines demonstrated the loss of epithelial polarity that weakened cell-to-cell contact in tumor cells that favored the ability to metastasize more easily [46]. This places the observed differential expression of the protein across the three phenotypes in the right perspective.

#### 3.3.7. WD Repeat-Containing Protein 1 (WDR1)

WDR1, also known as actin-interacting protein 1 (AIP1), interacts with actin-depolymerizing factor (ADF)/cofilin to accelerate actin filament disassembly [47]. In our study, we found a high expression of WDR1 in leukoplakia and furthermore in cancer, as compared to the control. WDR1 plays a crucial role in mammalian cell cytokinesis and chemotactic migration by enhancing lamellipodial actin dynamics which is an essential feature in cancer [48]. WDR1 enhances cancer progression by positively regulating cell migration and mitotic cell division [49]. This clearly explains that the 2.1-fold change difference of WDR1 in cancer is implicative in the increase in intraepithelial proliferation initiating at leukoplakia that increases furthermore in cancer with migratory potential.

Cancer cells (1) have metabolic requirements such as increased glucose uptake that are met by glycolysis, (2) encounter higher oxidative damage due to increased reactive oxygen species production, and (3) show disruption in the cytoskeleton assembly and dynamics, facilitating motility and an aggressive nature. In summary, the malignant transformation of oral precancerous lesions to cancer is primarily dictated by increased glycolysis-mediated metabolism, response to oxidative stress, and cytoskeletal disruption.

The mass spectrometry proteomic data has been deposited at the ProteomeXchange Consortium via the PRIDE partner repository with the dataset identifier PXD037660 [50].

### 3.4. Validation

Bioinformatic analysis was done to functionally annotate the proteins to understand their role in biological processes and pathways that they are involved in. Unsupervised clustering approaches like hierarchical clustering, heat map, and principal component analysis were performed to discover patterns and subgroups in the data so as to visually identify relationships or similarity between the samples in terms of the proximity of the points.

**Figure 4:**
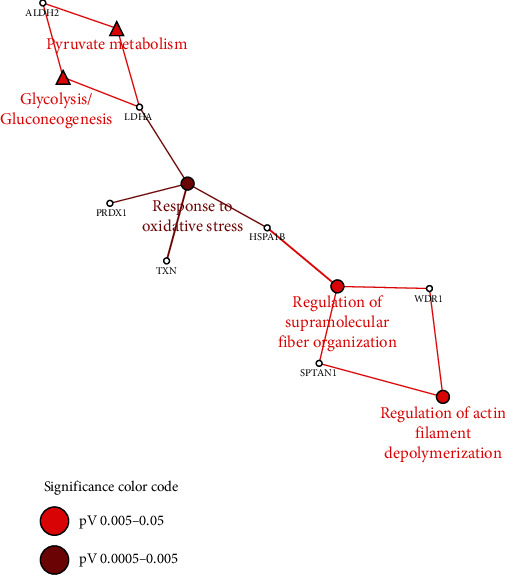
Functional annotation of differentially expressed proteins with biological functions and pathways. TXN, PRDX1, HSPA1B, and LDHA are involved as a response to oxidative stress. SPTAN1 and WDR1 regulate actin filament depolymerization. LDHA and ALDH2 are involved in carbon metabolism pathways such as glycolysis and pyruvate metabolism.

Seven differentially expressed proteins were recognized for functional annotation. The main biological processes and KEGG pathways associated with our study of epithelium transformation are listed in Table 5 and Figure 4. TXN, PRDX1, HSPA1B, and LDHA are seen to be involved as a response to oxidative stress. It may be recalled that the same four proteins have a regulation relating to oxidative stress that has been detailed in previous paragraphs. SPTAN1 and WDR1 regulate actin filament depolymerization. The LDHA and ALDH2 were found to be involved in carbon metabolism pathways including glycolysis and pyruvate metabolism. These mechanisms require a gradual increment of the involved proteins along control, precancerous lesions, and cancer. Therefore, the functional association of differentially expressed proteins as presented by the bioinformatic study correlates well in context with functions of proteins which have previously been discussed in the discovery phase of proteomics in terms of progression of the epithelium from a precancerous lesion to cancer.

**Figure 5:**
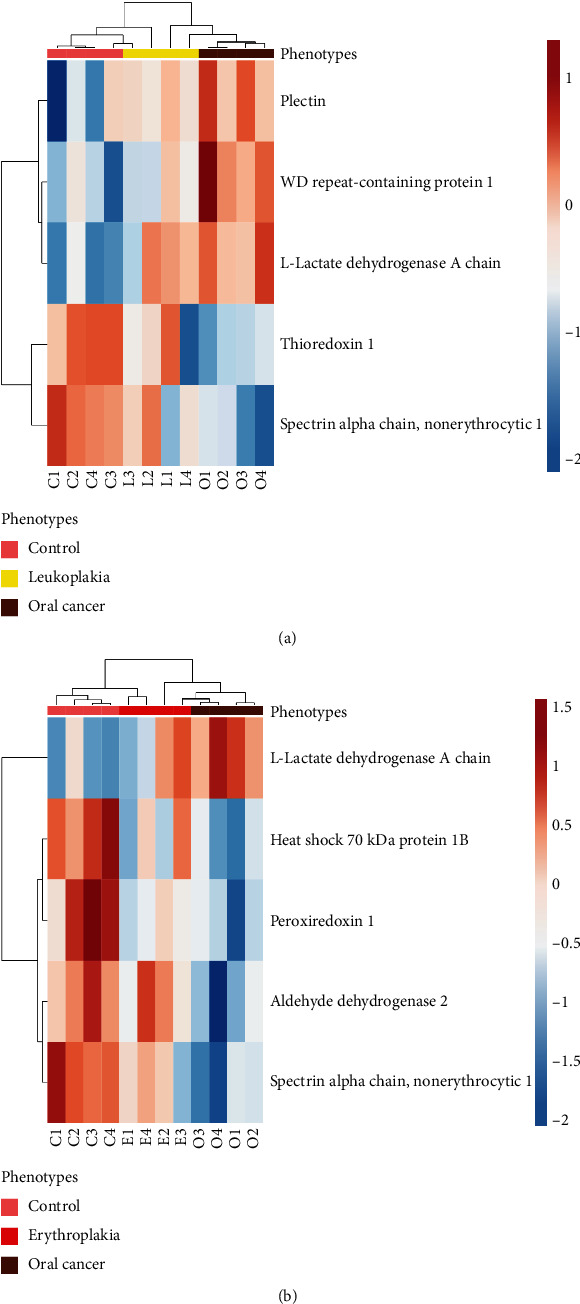
Hierarchical clustering and heat map showing relative protein intensities of differentially expressed proteins. (a) Control, leukoplakia, and cancer; (b) control, erythroplakia, and cancer. Red corresponds to overexpression and blue to underexpression of the protein.

A two-way hierarchical clustering heat map display showed the protein expression across phenotypes in each sample used in the study (Figure 5). Considering column-based clustering of phenotypes, in both figures, the two main clusters are seen getting subclustered further. The first cluster is comprised of 4 control samples and 2 precancerous samples whereas the second cluster is comprised of 2 precancerous and 4 cancer samples. We observed one leukoplakic sample very similar to the control whereas the other 3 leukoplakia samples diverged into separate branches from their respective main clusters. Similarly, two erythroplakia samples diverged into a separate cluster, and the other two showed similarities to cancer groups. Visualizing expression patterns in a heat map, we identified a gradual increase or decrease in the protein expression across phenotypes as well as an overlapping expression of precancerous lesions with that of the control or cancer. These protein expressions across phenotypes validate the discovery phase results.

**Figure 6:**
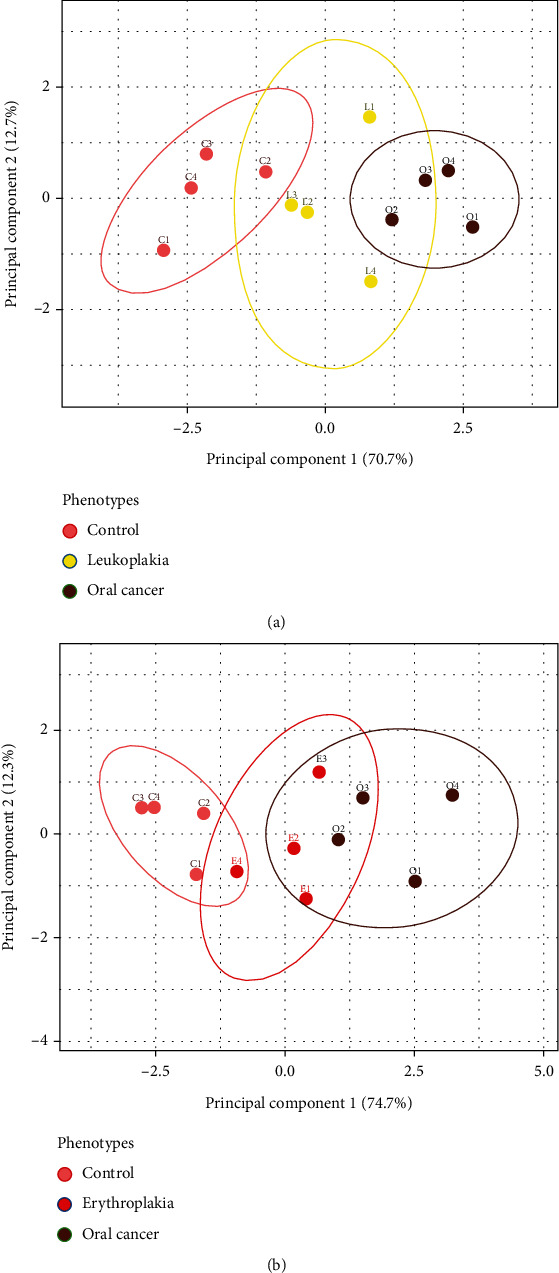
Principal component analysis of differentially expressed proteins across (a) control, leukoplakia, and cancer; (b) control, erythroplakia, and cancer. Control and cancer phenotypes form well-segregated distinct cluster, whereas erythroplakia phenotypes show higher overlap with the cancer cluster, as compared to leukoplakia.

Principal component analysis (PCA) projection for proteins involving leukoplakia and erythroplakia explained a minimum of 70% variance across principal components (Figure 6). Cancer and control phenotypes were found to be well segregated clusters as compared to leukoplakia. However, there were certain overlaps between control vs. leukoplakia and leukoplakia vs. cancer. Half of the leukoplakic samples showed close proximity to the control, whereas the other half was towards cancer. Similarly, we found three erythroplakia samples overlapped with cancer samples, and one overlapped with the control. These findings indicate that erythroplakia is at a higher propensity to turn malignant, as compared to leukoplakia.

## 4. Conclusion

Label-free proteomics offers a good platform for the identification of protein biomarkers that can flag premalignant conditions that could become malignant. Qualitative and quantitative protein signatures across control, precancer, and cancer phenotypes explain the possible functional outcomes that dictate malignant transformation to oral carcinoma. The observations place the previously observed clinical findings of erythroplakia having more predilection for cancer, in the right perspective.

## Figures and Tables

**Table 1:** Clinicopathological profile of patients recruited for the study.

S. no.	Patient ID	Age/gender	History of tobacco consumption	Tissue description	Histopathology	Clinical phenotype
1	B0L19	36 y/M	+	Normal-looking tissue adjacent to whitish lesion on right buccal mucosa	Hyperplasia	Cancer control
2	AHL19	58 y/M	+	Normal-looking tissue adjacent to whitish lesion on right buccal mucosa	Hyperplasia	Cancer control
3	NKU19	52 y/M	+	Normal-looking tissue adjacent to whitish lesion on left buccal mucosa	Mild dysplasia	Cancer control
4	DSL20	63 y/M	+	Normal-looking tissue adjacent to whitish lesion on right buccal mucosa	Hyperplasia	Cancer control
5	C0L19	44 y/M	+	Whitish lesion over right side of buccal mucosa	Dysplasia	Leukoplakia
6	R0L19	65 y/F	+	Homogenous whitish lesion over right side of buccal mucosa	Dysplasia	Leukoplakia
7	NKL19	52 y/M	+	Whitish lesion on left side of buccal mucosa	Dysplasia	Leukoplakia
8	A0L19	36 y/M	+	Indurated whitish lesion over right side of buccal mucosa	Dysplasia	Leukoplakia
9	SCE20	44 y/M	+	Reddish lesion on left side of buccal mucosa	Dysplasia	Erythroplakia
10	G0E20	58 y/F	+	Reddish lesion on left side of buccal mucosa	Dysplasia	Erythroplakia
11	SGE19	45 y/M	+	Reddish lesion on left side of buccal mucosa	Dysplasia	Erythroplakia
12	R0E19	42 y/F	Information not available	Reddish lesion on left side of buccal mucosa	Dysplasia	Erythroplakia
13	SAC19	58 y/M	+	Ulceroproliferative growth on right side of buccal mucosa	Neoplasia	Squamous cell carcinoma
14	KFC19	71 y/F	+	Ulceroproliferative growth on left side of buccal mucosa	Neoplasia	Squamous cell carcinoma
15	NAC20	48 y/M	+	Ulceroproliferative growth with induration on left side of buccal mucosa	Neoplasia	Squamous cell carcinoma
16	MKC20	42 y/M	Information not available	Ulceroproliferative growth on right side of buccal mucosa	Neoplasia	Squamous cell carcinoma

**Table 2:** Demographic features of the patient cohort.

Phenotypic groups	Gender		Mean age (in years)
	Male	Female	
Control	4	—	52 (52.25 ± 11.7)
Leukoplakia	3	1	49 (49.2 ± 12.3)
Erythroplakia	2	2	47 (47.2 ± 7.2)
Oral cancer	3	1	55 (54.75 ± 12.6)

**Table 3:** Differentially expressed proteins in control, leukoplakia, and cancerous phenotypes.

S. no.	Protein	Function	Relevance in our study	References
1	LDHA (L-lactate dehydrogenase A) chain	Facilitates glycolysis	Provides higher energy to cells for proliferation and survival bypassing oxidative phosphorylation thereby protecting cells from ROS damage	[29–33]
2	TXN (thioredoxin)	Maintain cellular redox homeostasis-protecting cells from ROS-induced damage	Decreased antioxidant activity results in endogenous ROS accumulation causing oxidative stress build-up that results in genomic instability promoting cellular proliferation	[36–39]
3	PLEC (plectin)	Acts as a cytoskeleton linker and scaffold-signaling protein that stabilize intermediate filament networks in the cell	Increased during higher amount of cellular proliferation, migration, and invasion in cancer cells	[42–44]
4	SPTAN1 (spectrin alpha chain, nonerythrocytic 1); fodrin	Acts as a scaffolding protein that maintains cell polarity and cell-to-cell contact	Reduced expression resulting in weaker cell-cell interaction resulting in detachment and metastasis of cancer cells	[45–46]
5	WDR1 (WD repeat-containing protein 1)	Promotes cofilin-mediated actin filament assembly	High amount of it in cancer enhances lamellipodial actin dynamics promoting cellular migration and cell division	[47–49]

**Table 4:** Differentially expressed proteins in control, erythroplakia, and cancerous phenotypes.

S. no.	Protein	Function	Relevance in our study	References
1	LDHA (L-lactate dehydrogenase A) chain	Facilitates glycolysis	Provides higher energy to cells for proliferation and survival bypassing oxidative phosphorylation thereby protecting cells from ROS damage	[29–33]
2	ALDH2 (aldehyde dehydrogenase 2)	Metabolizes aldehydes	Aggressive behaviours like migration and invasion were inversely related to ALDH2 expression	[34–35]
3	PRDX1 (peroxiredoxin 1)	Maintains cellular redox homeostasis-scavenging peroxides	Decreased antioxidant activity results in endogenous ROS accumulation causing oxidative stress build-up that results in genomic instability promoting cellular proliferation	[36–39]
4	HSPA1B (heat shock 70 kDa protein 1B)	Stress-inducible molecular chaperone	Higher expression of heat shock proteins enhanced the resistance of cells to apoptosis resulting in countering hypoxic stress and enhancing cell survival	[40–41]
5	SPTAN1 (spectrin alpha chain, nonerythrocytic 1); fodrin	Acts as a scaffolding protein that maintains cell polarity and cell-to-cell contact	Reduced expression resulting in weaker cell-cell interaction resulting in detachment and metastasis of cancer cells	[45–46]

**Table 5:** Functional association of proteins assessed using ClueGO+CluePedia.

S. no.	Term	Ontology source	Term *p* value corrected with Bonferroni step-down	Associated genes (%)	Associated genes found
1	Response to oxidative stress	GO_biological process	1.9*E* − 3	0.84	[HSPA1B, LDHA, PRDX1, TXN]
2	Regulation of supramolecular fiber organization	GO_biological process	3.2*E* − 2	0.75	[HSPA1B, SPTAN1, WDR1]
3	Regulation of actin filament depolymerization	GO_biological process	1.7*E* − 2	3.45	[SPTAN1, WDR1]
4	Glycolysis/gluconeogenesis	KEGG	2.2*E* − 2	2.99	[ALDH2, LDHA]
5	Pyruvate metabolism	KEGG	1.1*E* − 2	4.26	[ALDH2, LDHA]

## Data Availability

The mass spectrometry proteomic data have been deposited at the ProteomeXchange Consortium via the PRIDE partner repository with the dataset identifier PXD037660.

## Supplementary Materials

Supplementary Materials Hematoxylin and eosin-stained histopathology sections of biopsies from sample phenotypes. (A, B, D) are biopsies from control tissues showing epithelial hyperplasia; (C) is a biopsy from control tissue showing dysplasia; (E–H) are biopsies from leukoplakic lesions showing epithelial dysplasia; (I–L) are biopsies from erythoplakic lesions showing epithelial dysplasia; and (M–P) are biopsies from neoplastic lesions showing squamous cell carcinoma.
