# Supplementary figures and images for: Label-Free Proteomics of Oral Mucosa Tissue to Identify Potential Biomarkers That Can Flag Predilection of Precancerous Lesions to Oral Cell Carcinoma: A Preliminary Study
Source: Dis Markers. 2023 Feb 1;2023:1329061. doi: 10.1155/2023/1329061 (PMC9908334; doi:10.1155/2023/1329061)

## Slide 1
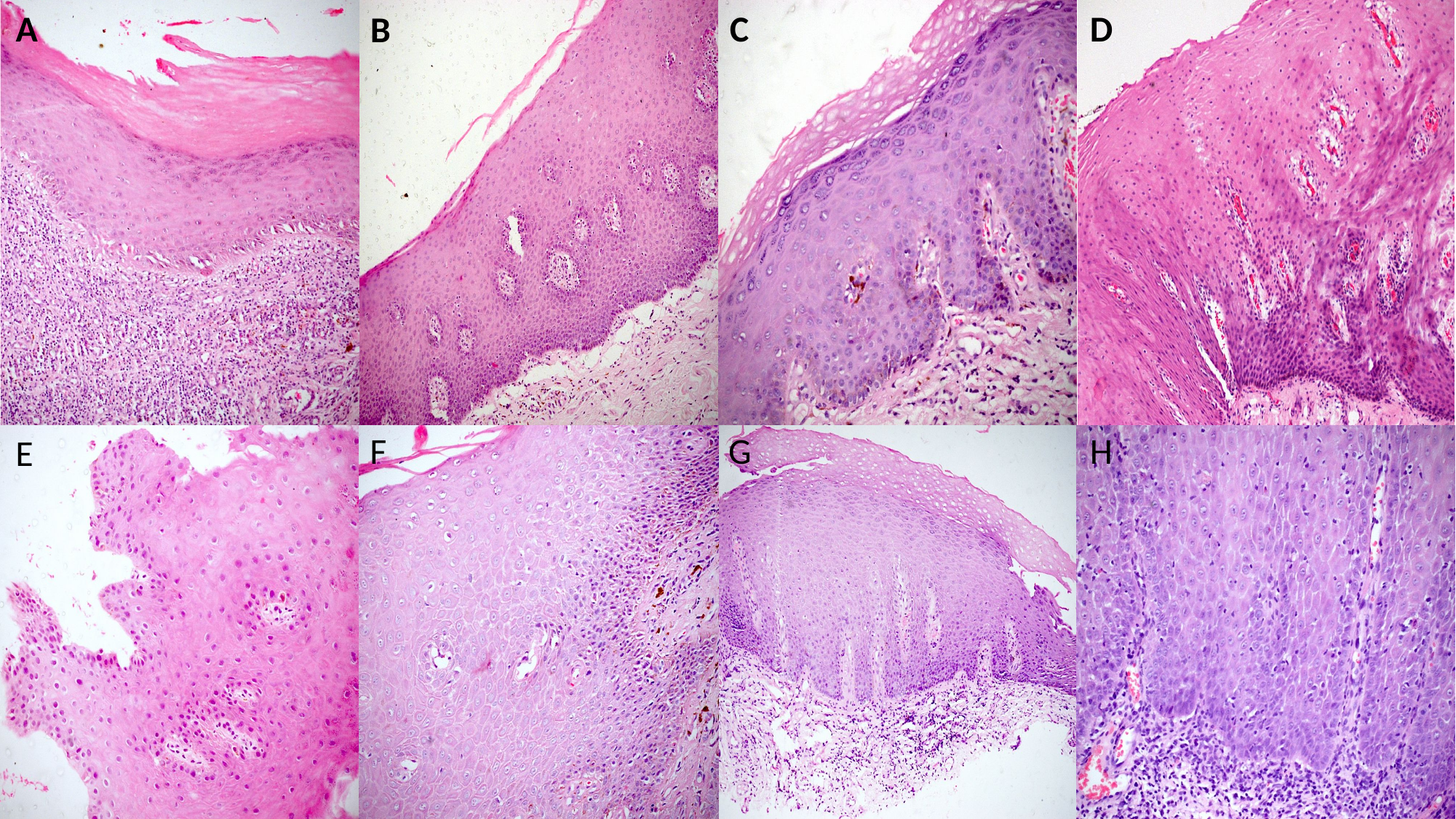

A
C
D
B
F
G
H
E

## Slide 2
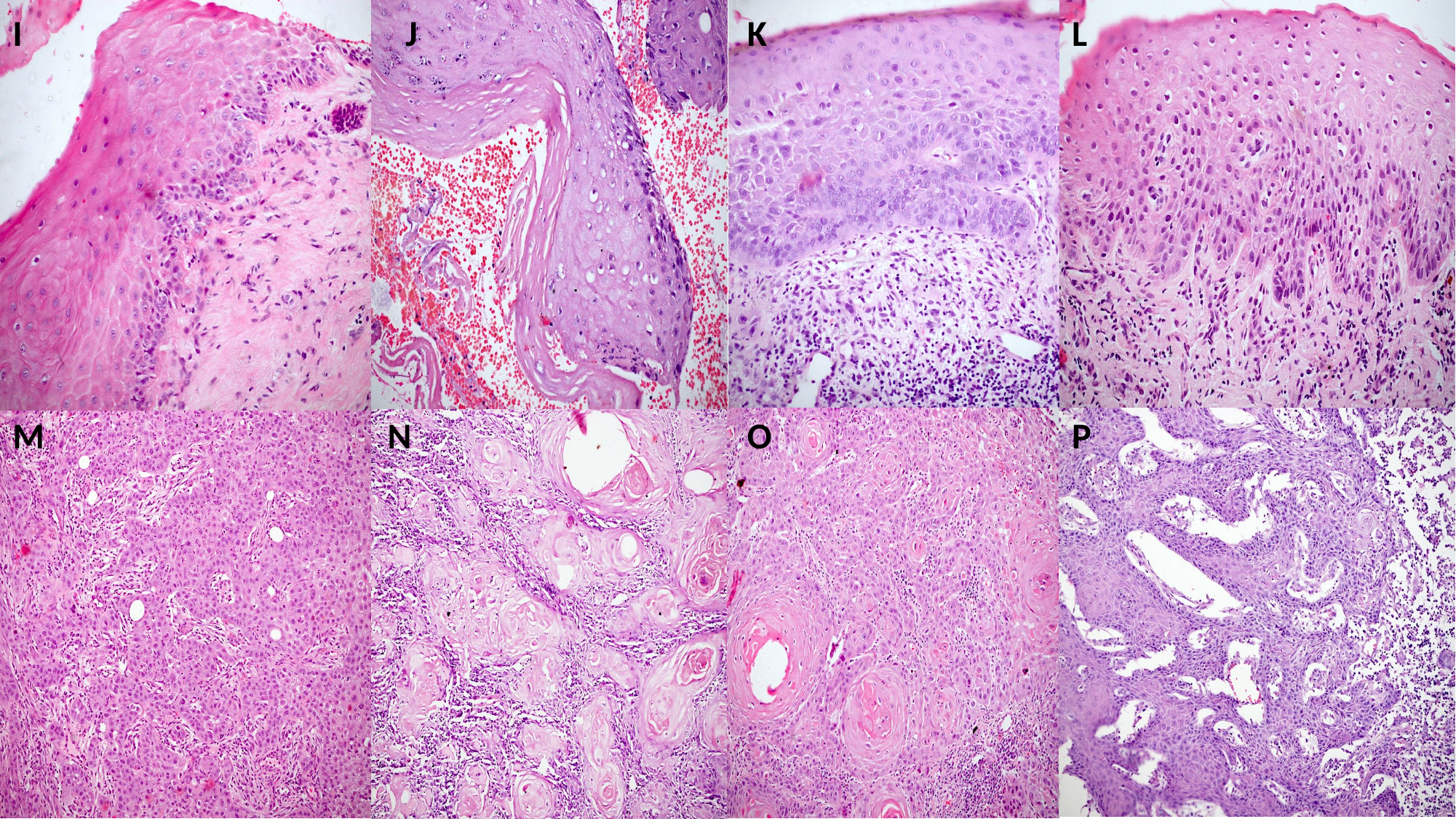

A
C
D
B
F
G
H
E
I
J
K
L
M
N
O
P

Supplement: Supplementary Materials — Hematoxylin and eosin-stained histopathology sections of biopsies from sample phenotypes. (A, B, D) are biopsies from control tissues showing epithelial hyperplasia; (C) is a biopsy from control tissue showing dysplasia; (E–H) are biopsies from leukoplakic lesions showing epithelial dysplasia; (I–L) are biopsies from erythoplakic lesions showing epithelial dysplasia; and (M–P) are biopsies from neoplastic lesions showing squamous cell carcinoma. [file 1329061.f1.pptx]
